# The single individual in medicine: how to escape from the probability theory trap

**DOI:** 10.1186/1757-1626-1-58

**Published:** 2008-07-24

**Authors:** Enzo Grossi

**Affiliations:** 1 Dipartimento Farma Italia, Bracco S.p.A, Via XXV Aprile, 420097 San Donato Milanese, Italy

## Abstract

Doctors and their patients are always concerned with the likely outcome of an existing disease and the risk of future diseases, but there are many problems in interpreting for the individual data derived from populations. Yet recent developments in mathematics and science should allow us to do much better.

## Editorial

An individual patient is not the average representative of the population. Rather he or she is a person with unique characteristics. An intervention may be effective for a population but not necessarily for the individual patient. The recommendation of a guideline may not be right for a particular patient because it is not what he or she wants, and implementing the recommendation will not necessarily mean a favourable outcome.

Clinical epidemiology and medical statistics are not suited to answer specific questions at the individual level. They focus on groups of individuals and not on single individuals. Classical statistics by definition needs samples to work, and samples by definition are always greater than one. This explains why is it almost impossible to perform any kind of statistics in the single individual. But despite these limitations physicians are forced to transfer statistical concepts emerging from groups to single individuals with deficiencies that the patient (and even the doctor) do not always understand. As Michael Kattan wrote in a famous editorial the patient who is in front of us may increasingly often say: "I am a patient, not a statistic!" [1].

Evidence based medicine, which is a natural product of classical statistics and randomized clinical trials, has led to the development of management protocols that are the best compromise for a group of patients defined in a certain way. But when abstract guidelines "hit" real patient care experience clearly shows that (with very few exceptions) no protocol fits every patient; and, more importantly, no protocol fits any patient perfectly.

Clinicians need measures of outcomes among individual patients during a trial as well as during routine clinical activity, especially when they expect considerable variation in the outcome. A confidence interval is inadequate for the clinician to decide what the useful the results of a megatrial will mean in clinical practice. Average results and confidence intervals from megatrials conceal huge diversity among the results for individual subjects.

The narrow confidence intervals generated by megatrials (and even more so by meta-analyses) are often understood to mean that doctors can be confident that the estimates of therapeutic effectiveness are valid and accurate. This is untrue both in the narrowly statistical sense as well as in the broad clinical sense. In fact the confidence intervals give no indication of the precision of an estimate for an individual in a trial. Furthermore, the narrowness of a confidence interval does not have any relation to a possible causal relation. Nor does it give any indication of the applicability of a trial result to another population. A resulting paradox is that narrow, non-overlapping confidence intervals that discriminate sharply between protocols in a statistical sense may, nevertheless, be associated with a variation among subjects such that some patients may be harmed by a treatment that benefits the majority.

We know that any kind of statistical inference is extremely weak in the absence of a "sample", which by definition requires a number greater than one. For this reason predictive models can fail dramatically when applied to the single individual. In a model that has an overall 90% accuracy in predicting an event on a group level, the degree of confidence can drop substantially when applied to a single subject.

Suppose that a predictive model for risk assessment in study data has been developed and validated and that it allows an overall accuracy of 0.9. Suppose that the confidence interval of this predictive rate is 0.06 (0.84–0.96). The first step is to assess a group of new subjects with our tools. We can reasonably expect to make classification mistakes within a range of 4%–16%. Therefore 4 to 16 of 100 new patients would be incorrectly assessed with regard to their absolute risk. If a new patient has been classified as at high risk to suffer from myocardial infarction in the next 10 years, the patient might think that there is a 90% chance that he or she has been correctly classified (84% at worst and 96% at best). Unfortunately the patient's confidence interval in this classification would not be equal to that of the group since with misclassification the patient could have either a correct prognosis or incorrect prognosis – a 100% difference.

One of the major challenges to delivering effective treatment is to devise a method capable of determining the confidence interval of a single individual.

This would be possible by feeding the data into artificial adaptive systems based on recursive algorithms that can solve problems that are not accessible to classical statistics. The findings from this new field of network mapping allow us to know what until now would have been science fiction. With a large data set it is theoretically possible to find individuals within the original population who are closest to our subject according to all descriptive parameters. This is feasible through non-linear mapping with specific evolutionary algorithms.

Taking into account all the descriptors available we should be able to match our subject with a suitable subgroup of subjects or even to another single subject. If we succeed in doing so we could take advantage of the statistics of a group to benefit the single individual. Unfortunately the problem is not simple. Suppose that the reference population is 1000 individuals and each individual is described by 50 variables. Finding a single individual who is most similar to the subject on study according to all variables means a non-polynomial time computation problem in which there are 360^48^possibilities to check. Even with the most powerful computer the time needed for such an analysis would be beyond any practical possibility.

The PST (Pick and Squash Tracking) is an evolutionary algorithm able to find the best spatial distribution of a given number of "objects" (in our case human subjects) described by a given number of variables. The number of subjects and variables can be very high, but despite this the algorithm is able to cope with explosive growing of dimensionality of the observation vector. The trick is to respect at maximum degree the "distances" of the objects among each other in the multidimensional space without exploring all the possible combinations but adaptively evolving through the optimal solution [2]. This avoids the so-called "curse of dimensionality". To be mathematically precise the PST algorithm carries out a multidimensional scaling from an N dimensional to a L dimensional space (where N>>L) and typically where L = 2 or L = 3. PST acts in this dimensional reduction to ensure that the original distance between points has the minimal amount of distortion in the L- dimensional space. In the case in which N< = L this distortion approaches zero. PST carries out this type of projection by means of an evolutionary algorithm called GenD [3], specifically defined to achieve this aim. Pilot experiences in medicine are already available and seem quite encouraging [4,5].

This is probably the point at which most doctors begin to feel uncomfortable with the mathematics, but for those who want more I've appended a mathematical explanation. But what all this means is that we begin to have a method to go beyond populations to individuals when advising patients. This will be a very important development.

## Appendix

From the mathematical point of view the problem can be presented in the following form: given K points *X *= {*x*_1_, ...., *x*_*k*_}, or their distances in a N-dimensional space, find the distribution of these points *Y *= {*y*_1_, ...., *y*_*k*_} in a L-dimensional space with L<N, so that we minimize the "difference" between the original distances and those in the projected space.

If we define:

the matrix of the map distances *Md*(*Y*): *Md*_*ij *_= *D*^*L*^(*y*_*i*_, *y*_*i*_),

the matrix of the original distances *Rd*(*X*): *Rd*_*ij *_= *D*^*N*^(*x*_*i*_, *x*_*j*_),

and a measure of the dissimilarity between the two matrices *E *= *E*(*Md*, *Rd*),

then the target function consists in finding a configuration of points *Y' *= {*y'*_1_, ...., *y'*_*N*_}, such that:

(1) E* = min [*E*(*Md*(*Y'*), *Rd*(*X*))]

Typically, the Error on the map, *Md*(*Y*), is calculated using the Euclidean distance:

(2) $$D^{L}(y_{i},y_{j}):D_{ij}=\sqrt{\sum_{k=1}^{L}{\left(y_{ik}-y_{jk}\right)}^{2}2};$$

This distance is dynamically adjusted during the Pst evolution, to minimize the Error Function of the problem( E* = min [*E*(*Md*(*Y'*), *Rd*(*X*))]).
